# Acute fatigue affects reaction times and reaction consistency in Mixed Martial Arts fighters

**DOI:** 10.1371/journal.pone.0227675

**Published:** 2020-01-31

**Authors:** Radim Pavelka, Vít Třebický, Jitka Třebická Fialová, Adam Zdobinský, Klára Coufalová, Jan Havlíček, James J. Tufano

**Affiliations:** 1 Faculty of Physical Education and Sport, Charles University, Prague, Czech Republic; 2 National Institute of Mental Health, Klecany, Czech Republic; 3 Faculty of Science, Charles University, Prague, Czech Republic; National Taipei University of Nursing and Health Sciences, TAIWAN

## Abstract

Mixed Martial Arts (MMA) is a multielement combat sport where fighters need to quickly react to an opponent’s movements under fatigued conditions. Research indicates that fast reaction time is important in many sports, but the effect of fatigue has shown negative, null, or even positive influences on reaction time. However, few studies have been conducted in a controlled setting, especially using MMA figthers, whose matches are frequently resolved in a split-second. Therefore, this study investigated whether acute neuromuscular fatigue affects reaction and movement times, and their consistency in MMA fighters (*N* = 45). Before and after an upper-body Wingate test, a simple visual reaction time task was completed. Results showed a significant negative effect of fatigue on the reaction times and their consistency, with longer reactions (1.5% change) and lower consistency (14.7% change) after the Wingate test. Further, greater amounts of fatigue during the Wingate test seemed to negatively affect the consistency of post-Wingate movement time. Due to cumulative fatigue and the dynamic nature of MMA, our data indicate that not only the decrements in aerobic and anaerobic power likely affect a fighter’s performance, but their reaction time and motor time may also be compromised during a fight.

## Introduction

Mixed Martial Arts (MMA) is a rapidly growing multielement combat sport [1,2] combining various fighting styles used in other combat sports and traditional martial arts. It employs a wide variety of techniques where opponents fight in a standing position, mostly relying on punches and kicks (similar to boxing, kickboxing, and Muay Thai), but also in a clinch or on the ground, where they wrestle and grapple (e.g. using techniques from Brazilian Jiu-Jitsu, Judo, Greco-Roman wrestling, and freestyle wrestling) [3]. Due to its combined and demanding nature, MMA can be defined as an acyclical aerobic-anaerobic dynamic-strength sport since bouts involve repeated explosive movements, near-maximal dynamic work, and periods of exerting static strength [4]. Depending on the level, promoters, and organizers of a tournament, the fights are divided into several 3- or 5-minutes rounds [5] during which opponents employ and maintain maximal or near-maximal efforts, which accumulate fatigue throughout the duration of the fight.

Fight performance is affected by various factors such as aerobic endurance [6–8], maximal strength, anaerobic capacity [9], body composition [10,11], and body proportions [12, but see 13], among others. Likewise, the ability to register, process, and appropriately react to an opponent’s actions is of crucial importance [14–16]. Therefore, the time to respond, the total time interval involving both premotor reaction times (RT; the time from stimulus onset to initiating adequate reaction) [17], and movement times (MT; the interval between the initiation of movement to the completion of particular movement) is imperative. Response time in sports can affect the athlete’s ability to optimize performance, focus, and make appropriate decisions [18]. In combat sports, particularly combat sports relying on striking blows with limbs, the ability to quickly react (RT) and employ adequate moves (MT) (together with techniques, tactics, fight strategy, and anticipation of an opponent´s intention) are considered among the key factors determining performance and success [e.g. 16,19] and any competitor who is capable of quickly processing information about the opponent’s actions and speedily responding to the actions is more effective when it comes to performing various types of motor behaviours [20,21].

Response time depends on numerous aspects such as the type and magnitude of stimulation, arousal state [18,19,22,23], participant’s age and sex [24–26], and level of fatigue [18,27,28]. When considering fatigue, the current body of research does not fully agree, as some studies have reported non-significant or mixed effects of isometric or isotonic exercise and induced fatigue on RT and MT [27]. Moderate and high-intensity exercise was reported not affecting RT of trained and non-trained participants [29]. Similarly, no significant changes in RT were observed at induced fatigue levels below or above lactate threshold of physical education students [30]. Likewise, in judo competitors, levels of serum lactate before and after a contest were not associated with RT [31]. In contrast, several other studies have described a positive effect of strenuous physical activity on RT. For instance, Kashihara and Nakahara [32] found that choice reaction times are improved after physical exercise with moderate intensity effort in comparison with a non-exercise period in amateur level athletes. Similarly, in a sample of elite volleyball players, the RT significantly improved in the first game set compared to the pre-game set [33]. Further, Malhotra et al. [18] reported the lowest RT immediately after moderate intensity exercise. Comparably, Levitt and Gutin [34] observed the lowest RT during moderate intensity exercise and the highest at near-maximal efforts. On the other hand, fatigue has been repeatedly found to increase both RT and MT in exercise close to maximal effort [19,25,35]. In Greco-Roman wrestlers [19,35] RT increased with a duration of a match and the highest RT were measured at a near-maximal performance intensity. In taekwondo practitioners, RT and roundhouse kick impact were negatively affected by fatigue [36]. Also, higher choice reaction times were observed during exercise-induced fatigue (especially by anaerobic and super-maximal intermittent exercise) [37].

During MMA fights, techniques are often performed after or during anaerobic efforts (e.g. clinching, grappling, strike exchanges) and fighters need to respond quickly under physically demanding situations. Ability to appropriately react (performing appropriate offensive and defensive actions) to the opponent’s movements with low RT and MT, while being physically fatigued, is of high importance. Moreover, not only the average RT and MT during a fight but the in/consistency in individual responses when being physically fatigued might be “the tipping point” of registering and responding on time to opponent’s swipes and attempts for submission or knockout. Although acoustic, vestibular, tactile or kinesthetic stimuli are involved in fast decisions during a fight, visual information is the most crucial. Like in many combat sports where matches are resolved in a split second limb or trunk movements, fighters need to quickly recognize visual stimuli in order to choose adequate response to avoid throwdowns or strikes as up to 37% of MMA fights are won via knockouts [38].

Previous research has shown that high and cumulative levels of acute fatigue negatively affect RT [19,35], while lower levels of physical activity seem to improve RT [18,34], although some studies have found no effect [29,31]. These mixed results could be due to variability in the type of RT tasks employed, selected study sample, methods of fatigue induction (e.g., physical contests, game sets, ergometers focusing on the fatigue of lower extremities), or notably the level of fatigue (ranging from moderate to near-maximal efforts). A further limitation of previous studies is a number of RT measures, as only mean reaction and MT are usually considered. Therefore, the purpose of the present study was to test whether acute neuromuscular fatigue induced in a standardized, lab-based setting using arm-cranked Wingate test affects RT, MT and their consistency on simple visual stimuli in a sample of MMA fighters.

## Methods

Data from this study are part of a larger project which investigates multimodal characteristics related to competition outcome in MMA [39]. All procedures were carried out in accordance with the Declaration of Helsinki, and the study was approved by the Institutional Review Board of the National Institute of Mental Health, Czech Republic (reference number 28/15). All measurements were performed at the Biomedicine Laboratory of the Faculty of Physical Education and Sport, Charles University, Czech Republic during Q2 2016 and Q3 2017.

### Participants

The participants were recruited via social media advertisements, leaflets distributed at local MMA tournaments and gyms, and with the help of the Mixed Martial Arts Association of the Czech Republic (MMAA). Only active Czech male MMA fighters aged between 18–40 yrs., and with at least two fights on a record were included in the study.

A total of 45 Czech male MMA athletes took part in the study. Participants’ age, years of MMA training, number of underwent MMA fights, body height, body weight, and BMI were recorded (for descriptive statistics, see Table 1). When compared with previous studies investigating the physical profile of MMA fighters and similar combat sports athletes [4,40–43], our sample does not differ in reported descriptive parameters, e.g.–age, body height and weight.

**Table 1:** Descriptive statistics.

		Mean	SD	Minimum	Maximum
**Sample descriptive**	Age (yrs.)	26.7	5.91	18	38
	MMA training (yrs.)	5.01	2.55	1	12
	MMA fights	7.89	5.81	2	28
	Height (cm)	179.9	6.88	165	193.8
	Weight (kg)	81.55	11.19	60.6	112.4
	BMI (kg/m^2^)	25.15	2.72	21.25	32.3
**Wingate test**	Peak anaerobic power output (W)	653.92	129.56	422	966
	Mean anaerobic power output (W)	506.76	86.86	293.1	712
	Total work performed (kJ)	15.21	2.61	8.8	21.4
	Decrease of performance (W)	292.1	100.16	102.2	521.6
	Fatigue index (%)	43.74	8.68	21.7	61.7
Reaction times*	Mean RT PRE (ms)	266.15	37.37	223.58	395.63
	Mean RT POST (ms)	270.18	42.79	211	435.79
	Mean MT PRE (ms)	115.01	27.91	71.38	167.5
	Mean MT POST (ms)	111.83	28.9	63.25	182.67

* all values reported are after excluding outliers, see section Statistical analysis.

All participants were informed about the study goals, approved their participation by providing written informed consent, and were reimbursed with 400 CZK (app. 17 USD) for their participation in the whole project.

### Procedures

**Fig 1:**
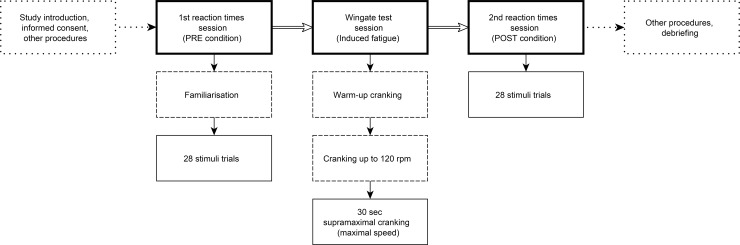
Flow chart of the data collection procedure.

We used a within-subject experimental design to test the effect of induced acute fatigue on RT, MT and their consistency. After familiarization with the data collection procedures, participants performed the first—a baseline (PRE condition)—RT test. To induce fatigue, participants completed a 30-seconds supramaximal anaerobic arm-cranking exercise at maximal speed against a frictional resistance by Wingate test which was immediately followed by the second RT test (POST condition) (Fig 1).

### Reaction and movement time measurements

The Vienna Test System (VTS-6; Schuhfried GmbH, Moedling, Austria) [for review see 44] running simple reaction time test (RT S1) was used to assess repeated RT and MT following a simple visual stimulus (a yellow circle appearing on a screen). During the RT S1, participants begin with the index finger of their dominant hand resting on a touch-sensitive rest button located on the VTS-6 response panel. At the onset of a stimulus, the participant lifts the index finger from rest button (RT) and move the finger as quickly as possible to a reaction button (MT) which is located above the rest button. The finger must then be returned to the rest button and must remain on the rest button until the subsequent stimulus. This procedure continues for the duration of the test, which consists of 28 visual stimuli, each of which appears on the screen maximally for 1 second in which the participant is supposed to respond. The length of pause between each stimulus varies (between 2.5 and 6.5 s) and is determined by the RT S1 program to avoid possible learning of the sequence. Depending on the speed of each participant, the test lasted on average 4 to 5 minutes.

Before the baseline test (PRE condition), participants completed a familiarisation run in which they learned how to perform the task and responded correctly to an abbreviated practice version of the test. If needed, feedback was given to help participants understand and complete the practice test correctly. After performing the familiarisation trial and confirming that they understood the test, the actual PRE test began. The POST condition test did not include any re-familiarisation.

Values of RT and MT and their variance are generated by the RT S1 test program. More specifically, they include the mean reaction time (RT; the time between the onset of the visual stimulus and the initial moment lifting of the finger from the rest button), the mean movement time (MT; the time between the finger leaving the rest button and touching the reaction button), and the variance in RT and MT times as a measure of performance consistency. For descriptive statistics, see Table 1. Further, raw RT and MT values were recorded for each stimulus, together with the number of correct responses (lifting a finger from the rest button and touching the response button), the number of incomplete responses (lifting a finger from the rest button but not touching the response button), and the number of failed responses (not lifting the finger from rest button). In the subsequent analysis, we used the individual raw RT, and MT and variance values recorded for each of 28 stimuli.

### Wingate test

An upper-body Wingate test was used to induce acute neuromuscular fatigue via a Monark arm ergometer (model Rump-Rokos 4.00/C01) [e.g. 45]. The test consisted of 30 seconds supramaximal arm-cranking exercise at maximal speed against the frictional resistance of 4 W×kg^-1^ determined relative to the participant’s body weight [46,47]. While seated on a bench, participants warmed up by cranking against no resistance until the heart rate reached 120 beats per minute. After that, participants started to crank as quickly as possible until they reached 120 revolutions per minute, which was followed by activation of the load and participants were verbally encouraged to perform at maximal turning rates throughout the 30-second period.

Several variables can be measured as outcomes of the Wingate test like peak anaerobic power output or total work performed, among others. Here, to assess a relative decrease in performance (fatigue), we focused only on the percentage of power decrease during the test (Fatigue index) in further analyses. For descriptive statistics, see Table 1.

### Statistical analysis

All statistical tests were performed using jamovi 1.0.7 [48]. The results of statistical tests with *p* values ≤ 0.05 were considered significant.

After an initial data exploration of the PRE RT values of all 28 stimuli, we found that the RT of the first four stimuli showed higher means and SD compared to the rest of subsequent stimuli, despite previously performed familiarisation trial (for details, see the Supplementary materials). Therefore, we excluded these first four stimuli from all subsequent analysis, leaving data from the remaining 24 stimuli. This approach resulted in a total of 2160 individual responses (45 participants reacted to 24 stimuli at 2 time points). Further, failed (n = 1 PRE, n = 2 POST) and incomplete (n = 4 PRE, n = 3 POST) responses were excluded. Thus the final sample consisted of 2150 individual responses.

The differences in the RT and MT before and after the Wingate test were analysed with linear mixed-effects models using GAMLj jamovi module [49]. We used this analytical approach to account for variation in the level of the individual participants and to avoid potential bias due to the data aggregation (i.e. comparing mean values). We set both models (using REML fit) with RT and MT as the dependent variables, Condition (PRE × POST) as a factor, Fatigue index (FI), and the number of the stimuli (1–24) as covariates. Participant ID was set as a random intercept; Condition, Stimulus, FI and interaction Condition: Stimulus were set as Fixed Effects (modelRT <- lmer(RT ~ 1 + Condition + Stimulus + FI + Condition:Stimulus + (1|ID)). Further, using R (R x64 3.5.3 [50] via RStudio 1.2.1335 [51]; for the R script, see the Supplementary materials) absolute values of RT and MT residuals were extracted to assess performance consistency and used as dependent variables in two subsequent models exploring the performance consistency in RT and MT. The proportions of reduced error (pseudo R^2^) are reported as R^2^ Marginal (R^2^_M_, the variance explained by the fixed effects over the total variance of the dependent variable) and R^2^ Conditional (R^2^_C_, the variance explained by the fixed and the random effects together over the total variance of the dependent variable).

## Results

### Differences in reaction times and their consistency before and after the induced acute fatigue

Overall, the fixed effects of the RT model explained only small portion of the total dependent variable variance (R^2^_M_ = 0.004), while the fixed and random effects together explained close to 50% of the total dependent variable variance (R^2^_C_ = 0.482). POST RTs were found significantly higher than PRE (mean difference = 4.031 ms, SE = 1.726, *t*_2102_ = 2.335, *p*_Bonferroni_ = 0.02) (Fig 1). The stimulus number covariate was found close to formal level of significance (*p* = 0.066) showing a negative slope (RT improved with every stimulus). There was no significant effect of fatigue index and the Condition:Stimulus interaction. Fixed effect parameters estimates for the RT model are reported in Table 2. Random effects in RT showed large variation between participants (RT variance = 1478.841, SD = 38.456, ICC = 0.480).

**Table 2:** Summary of the fixed effects parameter estimates for RT and RT residuals models.

	Names	β	SE	95% CI		df	*t*	*p*
				Lower	Upper			
**Reaction times**	(Intercept)	266.154	5.861	254.666	277.641	44.93	45.41	< 0.001
	Condition	4.031	1.726	0.647	7.414	2102.015	2.335	0.02
	Stimulus	-0.324	0.176	-0.669	0.022	2102.009	-1.837	0.066
	Fatigue index	-0.261	0.675	-1.585	1.063	43.003	-0.386	0.701
	Condition ✻ Stimulus	0.058	0.249	-0.431	0.546	2102.015	0.231	0.818
**Reaction times residuals**	(Intercept)	0.477	0.031	0.416	0.537	53.896	15.465	< 0.001
	Condition	0.07	0.02	0.031	0.11	2101.87	3.46	< 0.001
	Stimulus	-0.003	0.002	-0.007	0.001	2101.835	-1.24	0.215
	Fatigue index	-0.005	0.003	-0.011	0.002	42.805	-1.403	0.168
	Condition ✻ Stimulus	0.003	0.003	-0.003	0.008	2101.867	0.902	0.367

Further, in the model using RT residuals (the measure of RT consistency) with R^2^_M_ = 0.012 and R^2^_C_ = 0.141, we found significant (*p* < 0.001) effect of PRE (mean PRE RT consistency = 0.477, SE = 0.031) and POST (mean POST RT consistency = 0.547, SE = 0.031) condition. Performance was less consistent (higher residuals) in the POST condition (mean difference = 0.07, SE = 0.02, *t*_2102_ = 3.46, *p*_Bonferroni_ < 0.001) (Fig 1). Other covariates (fatigue index, Stimulus) and Condition:Stimulus interaction in this model were not significant. Fixed effect parameters estimates for RT residuals model are reported in Table 2. Random effects in RT residuals showed very low variation between participants (RT residuals variance = 0.033, SD = 0.183, ICC = 0.133).

### Differences in movement times and their consistency before and after the induced acute fatigue

**Fig 2:**
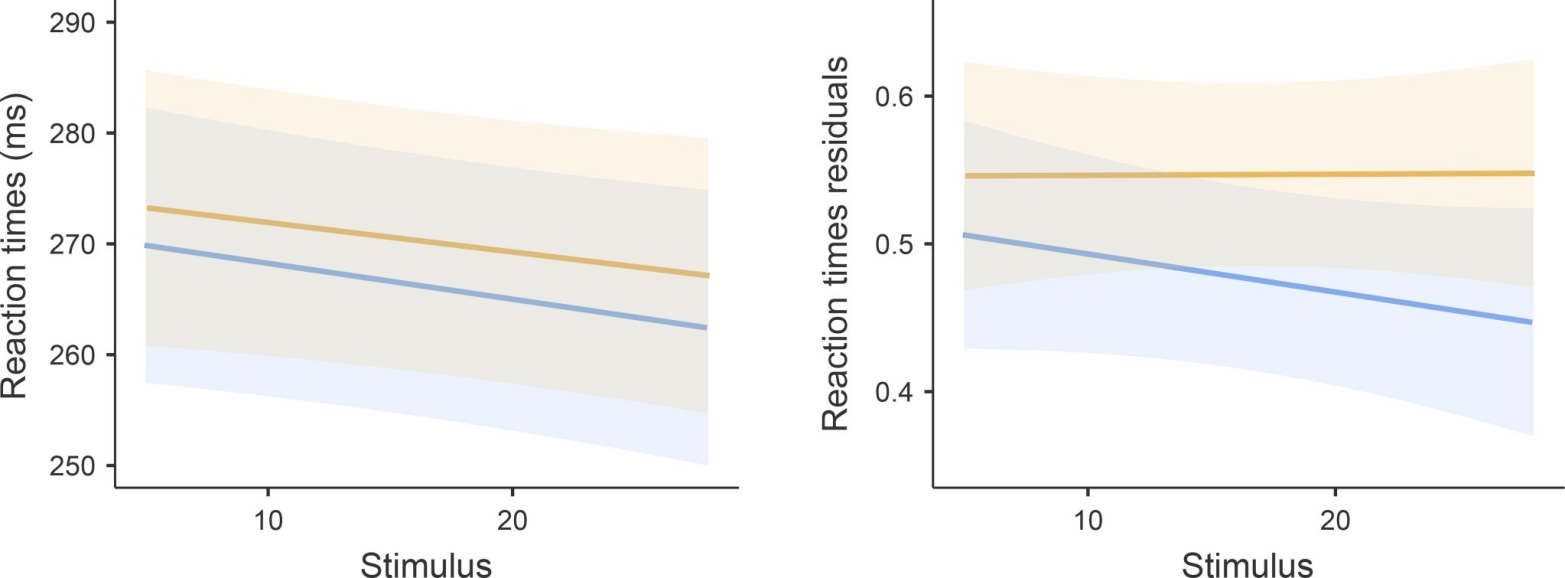
RT measures before and after the fatiguing task. Blue line represents the PRE condition, and the orange line represents POST condition for RT (left panel) and RT residuals (right panel). Semi-transparent areas indicate the 95% CI of the fixed effect of the difference in RT and RT residuals between PRE and POST the fatiguing task.

Both the fixed effects and the fixed and random effects together in the MT model explained only a small portion of the total dependent variable variance (R^2^_M_ = 0.003, R^2^_C_ = 0.035). We found no significant effect of the PRE× POST condition (Fig 2) or any of the covariates and Condition:Stimulus interaction on MT. The results thus show that mean MT remained uninfluenced by the fatiguing task. Fixed effect parameters estimates for the MT model are reported in Table 3. Random effects in MT showed moderate variation between participants (MT variance = 320.319, SD = 17.897, ICC = 0.032).

**Table 3:** Summary of the fixed effects parameter estimates for MT and MT residuals models.

	Names	β	SE	95% CI		df	*t*	*p*
				Lower	Upper			
**Movement times**	(Intercept)	115.008	4.02	107.129	122.888	82.729	28.606	< 0.001
	Condition	-3.18	4.253	-11.517	5.156	2102.256	-0.748	0.455
	Stimulus	-0.541	0.434	-1.392	0.31	2102.145	-1.247	0.213
	Fatigue index	0.21	0.398	-0.57	0.989	43.05	0.527	0.601
	Condition ✻ Stimulus	-0.315	0.615	-1.52	0.89	2102.246	-0.513	0.608
**Movement times residuals**	(Intercept)	0.38	0.038	0.306	0.455	75.817	9.983	< 0.001
	Condition	-0.013	0.038	-0.088	0.061	2102.028	-0.346	0.729
	Stimulus	-0.003	0.004	-0.01	0.005	2101.933	-0.659	0.51
	Fatigue index	0.008	0.004	0.0009	0.016	42.852	2.204	0.033
	Condition ✻ Stimulus	-0.007	0.005	-0.018	0.004	2102.019	-1.307	0.191

**Fig 3:**
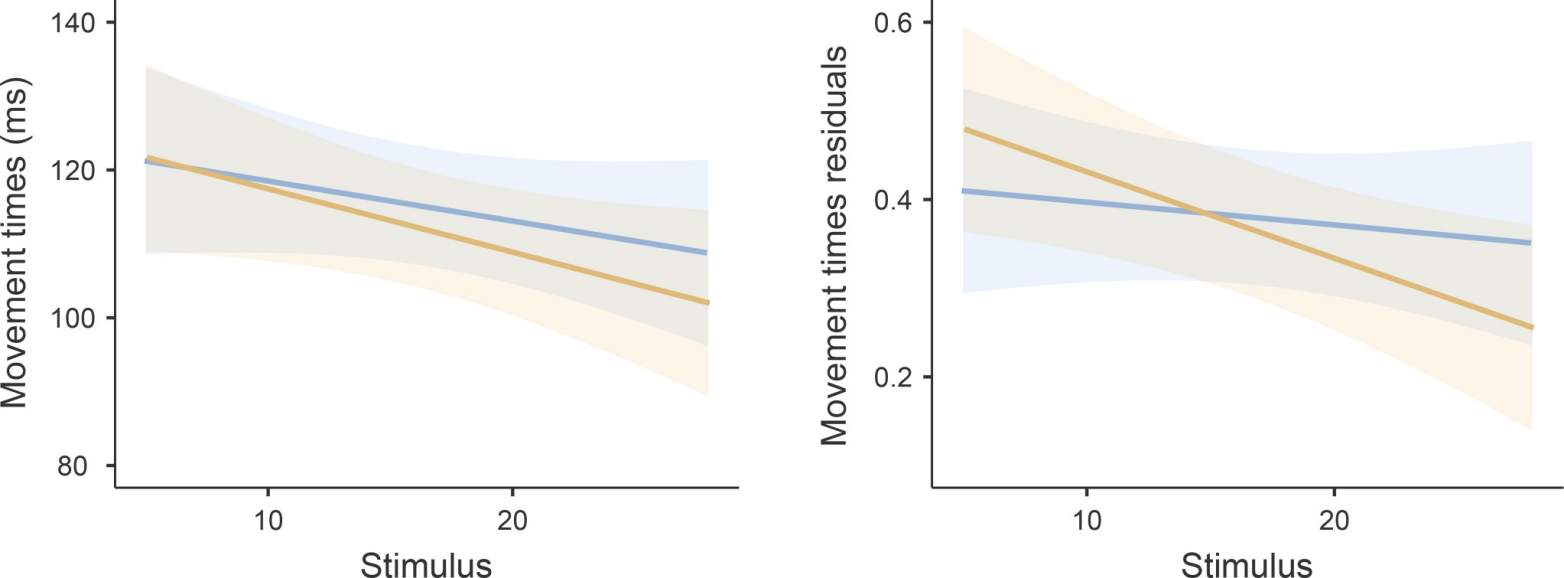
MT measures before and after the fatiguing task. Blue line represents the PRE condition, and the orange line represents the POST condition for MT (left panel) and MT residuals (right panel). Semi-transparent areas indicate the 95% CI of the fixed effect of the difference in MT and MT residuals between PRE and POST the fatiguing task.

In the model using MT residuals (the measure of MT consistency) with R^2^_M_ = 0.009 and R^2^_C_ = 0.05, we found a statistically significant ()positive effect (β = 0.008, SE = 0.004, *p* < 0.033) of fatigue index on MT residuals. There was no significant effect of CONDITION (mean RT residuals = 0.38, SE = 0.038) and POST (mean RT residuals = 0.367, SE = 0.038) on MT consistency (Fig 3). Further, the Stimulus and interaction between Condition:Stimulus were also not statistically significant. Fixed effect parameters estimates for MT residuals model are reported in Table 3. Random effects in MT residuals showed very low variation between participants (MT residuals variance = 0.033, SD = 0.183, ICC = 0.04).

## Discussion

In the present study, we tested the effect of induced acute fatigue on simple visual reaction time measures. For this purpose, we measured RT, MT and their consistency in a sample of male MMA fighters before and after they performed an upper-body Wingate test. Overall, we found that mean RT were significantly higher and less consistent (participants sometimes reacted slower and other times faster) in the POST compared to the PRE condition, no such changes were observed in mean MT and MT consistency. Further, the fatigue index during the Wingate test did not influence RT or their consistency, but a greater fatigue index was associated with slower MT in POST condition. These results thus suggest that acute fatigue affects overall performance and the consistency of simple RT measures in MMA fighters.

Several previous investigations showed similar significant effects of fatigue on overall response performance. With increasing exercise intensity, the reaction times become slower [30,36,52] as well as in case of near-maximal or super-maximal performance [19,25,35,37]. On the other hand, physical exercise of a moderate intensity was reported to affect participants’ reactions positively—decreasing RT [18,32,33], and the results of other studies reported no difference in mean reaction times before and after given exercise [29,31]. Differences in results regarding the effect of fatigue on response performance can thus be explained by the varying levels of induced fatigue and/or differences in exercises employed. In the current study, athletes performed standardized supramaximal arm-cranking exercise, and our results on both RT and MT are consistent with the line of research showing the negative effect of near-maximal effort and fatigue on performance in reaction time tasks. Interestingly, the majority of previous studies does not clearly distinguish between overall response performance and its components—RT and MT. Changes in both RT and MT in response to accumulated fatigue are thus not frequently described, or only overall performance or RT is reported [for an exception see 36].

Though the effect of PRE × POST condition on RT was significant in our study (although not in MT), this finding should be interpreted with some caution as the actual difference was of a rather marginal magnitude (only 4 ms, 1.5% increase). Interestingly, we found a close to significance positive effect of Stimulus number on RT. Participants tend to react faster with every stimulus, and as we found no interaction between Condition and Stimulus in the RT model, this pattern was consistent regardless of fatigue. Unlike previous studies, we included performance consistency (in the form of RT and MT residuals) among RT and MT measures. Our study thus bring new evidence of a negative influence of acute fatigue on RT and MT performance consistency. Individual participants’ RT were more variable in the POST condition (14.7% change), and the MT were more variable in individuals who showed higher fatigue.

MMA fights involve explosive and dynamic-strength work combining short periods of activity and rest. We thus employed short exercise consisting of 30 seconds of supramaximal work to assess the possible effect in a simple experimental setting. However, using only one such period might not represent sufficient load to elicit adequate fatigue comparable to the one experienced during the actual fight as active periods are repeated numerously during the whole fight. Studies by Gierczuk et al. [19,35] reported higher reaction times when testing Greco-Roman wrestlers after the second and third round of 3 round match only, but see the non-significant results in Judo [31] or Jiu-Jitsu [53] and non-significant results for MT and overall response performance in Taekwondo [36]. Future studies thus should use multiple repetitions of fatiguing tasks–or conditions in general similar to the actual competition—to better describe patterns in reaction times measures changes due to cumulative fatigue. Nevertheless, our results indicate that even relatively short exercise periods affect reaction times measures and their consistency. It remains an open question for further investigation, whether more accumulated fatigue would entail greater changes [19, 35].

In our study, we selected the arm-cranked Wingate test to induce fatigue. Arm-cranking exercise can be viewed as movements analogous to typical arm movements that are associated with combat sports relying on striking. Changes in performance due to induced fatigue through exercise can be explained by arousal theory [54] and multidimensional allocation of resources theory [55]. The amount of mental effort that individuals invest in a task and an increase in arousal accompanying physical exercise are factors determining resulting performance. At low and moderate arousal levels, the extra invested mental effort can compensate for performance losses and performance increases towards an optimal point. At very high arousal levels, performance is expected to decrease when the (limited) capacity for the effort is exceeded. The arousal encountered in the current study (elicited by the Wingate test) appeared sufficient and severe enough to affect mean RT and RT performance consistency. Such arousal thus influences overall performance including RT measurements based on movements of a finger.

Further, the Vienna system simple visual reaction times (RT S-1) test selected for this study might not seem like the most suitable measure due to MMA complexity or in comparison with other methods used to assess response performance in previous investigations (e.g. measures of muscle activation response via electromyography [36] or use of specialized reactometers [35]). MMA fight techniques consist of consecutively performed complex actions (series of holds, clinches, takedown attempts, and strike exchanges) which may call for more complex and selective reactions and decision making. In practice, fighters often perform quick single movements lasting fractions of seconds (knockout attempts, swipes) and they need to react swiftly in response to fast limb or trunk movements of an opponent. Usually, simple reactions are involved in response to such stimulus, and a well-trained contra-movement is applied. Hence simple reactions can be considered as the first level of automatic response preceding further and more complex processing of adequate reactions and in turn successful performance. Further, the in/consistency in individual reactions when fatigued (the pattern showed by our participants) might be the so far overlooked “tipping point” of registering and responding on time to opponent’s swipes and attempts for submission or knockout. We might observe such a pattern in many martial arts, and combat sport fights where matches are resolved in split-second inattentiveness or due to slower than desirable response.

To conclude, a short period of induced fatigue negatively affects performance in a simple visual reaction times task in a sample of MMA fighters. Our results thus extend the current knowledge about the adverse effects of fatigue on athletes’ performance [19,35,36] in combat sports. Among other important factors, changes in response performance might play a significant role in fighting success as fatigue negatively influences general performance and its consistency which might compromise appropriate and timely responses to opponent’s actions.

Successful performance in sports is affected by many physical and psychological factors such as strength, endurance, strategic thinking, or coping with stress. After a high-intensity effort, physical and mental fatigue may appear, and both may affect performance including ability to react quickly. This ability is probably highly important for combat sports athletes, where slower-than-desired reactions decrease chances to respond adequately and succeed in a match. Majority of studies usually investigate only the effect of averaged reaction times. Here we show evidence that not only mean reactions, but other reaction times measures are affected by fatigue differently. RT are negatively influenced by fatigue (though marginally in the case), more importantly RT consistency is lower, and fatigue index has negative effect on MT. Typically, the majority of attention is devoted to physical training including aerobic and anaerobic performance especially in combat sports such as MMA due to its dynamic and physically demanding nature. However, our results suggest that in such sports, where split-second errors cannot be afforded, the reaction times consistency might be more important criteria than previously considered. Future research and training programs thus should focus on reaction time consistency as a potential marker of general physical fitness and investigate the possibilities to prevent deterioration of athletes’ performance under fatiguing circumstances. This could lead to development of specialized training programs focused on improving fight strategy under fatiguing conditions.

## Supporting information

S1 Dataset (CSV)

S1 File Reaction times exploration. (XLSX)

S2 File Main analysis jamovi syntax. (PDF)

S3 File R script for residuals. (R)

S4 File (TXT)
