# Supplementary material for: Acute fatigue affects reaction times and reaction consistency in Mixed Martial Arts fighters
Source: PLoS One. 2020 Jan 31;15(1):e0227675. doi: 10.1371/journal.pone.0227675 (PMC6994193; doi:10.1371/journal.pone.0227675)
Supplement: S2 File — (PDF) [file pone.0227675.s003.pdf]

Mixed Model

Model Info

Info	
Estimate	Linear mixed model fit by REML
Call	RT ~ 1 + Condition + Trial + FI + Condition:Trial+(1|ID)
AIC	22125.943
R-squared Marginal	0.004
R-squared Conditional	0.482

Model Results

Fixed Effect Omnibus tests

	F	Num df	Den df	p
Condition	5.452	1	2102.015	0.020
Trial	5.588	1	2102.014	0.018
FI	0.149	1	43.003	0.701
Condition ✻ Trial	0.053	1	2102.015	0.818

Note. Satterthwaite method for degrees of freedom

Fixed Effects Parameter Estimates

				95% Confidence Interval				
Names	Effect	Estimate	SE	Lower	Upper	df	t	p
(Intercept)	(Intercept)	266.154	5.861	254.666	277.641	44.930	45.410	< .001
Condition1	2 - After - 1 - Before	4.031	1.726	0.647	7.414	2102.015	2.335	0.020
Trial	Trial	-0.324	0.176	-0.669	0.022	2102.009	-1.837	0.066
FI	FI	-0.261	0.675	-1.585	1.063	43.003	-0.386	0.701
Condition1 ✻ Trial	2 - After - 1 - Before ✻ Trial	0.058	0.249	-0.431	0.546	2102.015	0.231	0.818

Random Components

Groups	Name	SD	Variance	ICC
ID	(Intercept)	38.456	1478.841	0.480
Residual		40.018	1601.475	

Note. Numer of Obs: 2150 , groups: ID , 45

Post Hoc Tests

Post Hoc Comparisons - Condition

Comparison							
Condition		Condition	Difference	SE	test	df	$p_{bonferroni}$
1 - Before	-	2 - After	-4.031	1.726	-2.335	2102.012	0.020

Estimated Marginal Means

Condition

				95% Confidence Interval	
Condition	Mean	SE	df	Lower	Upper
1 - Before	266.154	5.861	44.930	254.348	277.959
2 - After	270.184	5.861	44.930	258.379	281.990

Note. Estimated means are estimated averaging across interacting variables

Trial

				95% Confidence Interval	
Trial	Mean	SE	df	Lower	Upper
Mean-1·SD	270.210	5.861	44.931	258.404	282.015
Mean	268.169	5.797	43.003	256.478	279.860
Mean+1·SD	266.128	5.861	44.931	254.323	277.934

Note. Estimated means are estimated averaging across interacting variables

FI

				95% Confidence Interval	
FI	Mean	SE	df	Lower	Upper
Mean-1·SD	270.408	8.198	43.004	253.875	286.941
Mean	268.169	5.797	43.003	256.478	279.860
Mean+1·SD	265.930	8.200	43.003	249.393	282.468

Note. Estimated means are estimated keeping constant other independent variable(s) in the model to the mean

Condition:Trial

					95% Confidence Interval	
Condition	Trial	Mean	SE	df	Lower	Upper
1 - Before	Mean-1·SD	268.394	5.987	48.912	256.362	280.425
2 - After	Mean-1·SD	272.026	5.987	48.908	259.994	284.058
1 - Before	Mean	266.154	5.861	44.930	254.348	277.959
2 - After	Mean	270.184	5.861	44.930	258.379	281.990
1 - Before	Mean+1·SD	263.914	5.986	48.890	251.883	275.945
2 - After	Mean+1·SD	268.343	5.988	48.930	256.310	280.376

Note. Estimated means are estimated keeping constant other independent variable(s) in the model to the mean

Fixed Effects Plots

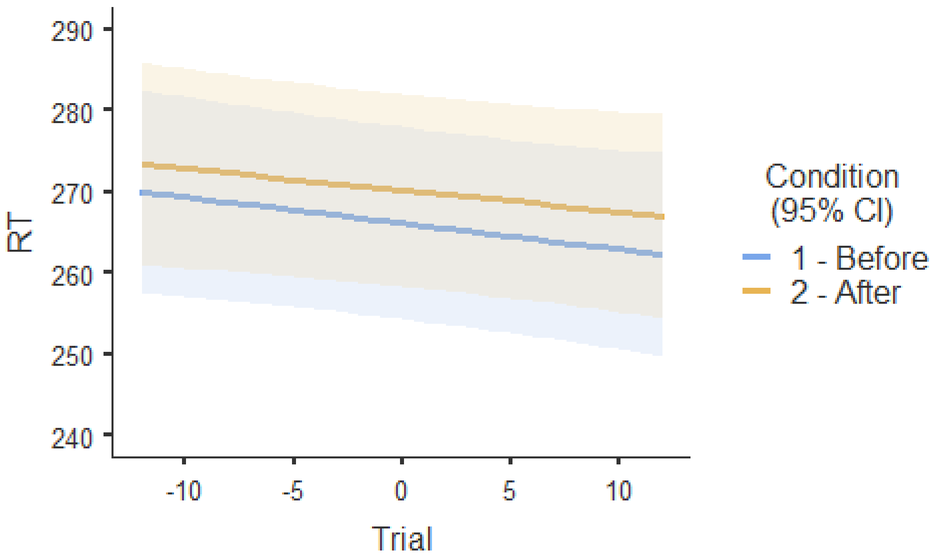

Mixed Model

Model Info

Info	
Estimate	Linear mixed model fit by REML
Call	MT ~ 1 + Condition + Trial + FI + Condition:Trial+(1|ID)
AIC	25871.410
R-squared Marginal	0.003
R-squared Conditional	0.035

Model Results

Fixed Effect Omnibus tests

	F	Num df	Den df	p
Condition	0.559	1	2102.256	0.455
Trial	5.173	1	2102.238	0.023
FI	0.278	1	43.050	0.601
Condition ✻ Trial	0.263	1	2102.246	0.608

Note. Satterthwaite method for degrees of freedom

Fixed Effects Parameter Estimates

				95% Confidence Interval				
Names	Effect	Estimate	SE	Lower	Upper	df	t	p
(Intercept)	(Intercept)	115.008	4.020	107.129	122.888	82.729	28.606	< .001
Condition1	2 - After - 1 - Before	-3.180	4.253	-11.517	5.156	2102.256	-0.748	0.455
Trial	Trial	-0.541	0.434	-1.392	0.310	2102.145	-1.247	0.213
FI	FI	0.210	0.398	-0.570	0.989	43.050	0.527	0.601
Condition1 ✻ Trial	2 - After - 1 - Before ✻ Trial	-0.315	0.615	-1.520	0.890	2102.246	-0.513	0.608

Random Components

Groups	Name	SD	Variance	ICC
ID	(Intercept)	17.897	320.319	0.032
Residual		98.604	9722.789	

Note. Numer of Obs: 2150 , groups: ID , 45

Post Hoc Tests

Post Hoc Comparisons - Condition

Comparison							
Condition		Condition	Difference	SE	test	df	$p_{bonferroni}$
1 - Before	-	2 - After	3.180	4.253	0.748	2102.210	0.455

Estimated Marginal Means

Condition

Condition	Mean	SE	df	95% Confidence Interval	
				Lower	Upper
1 - Before	115.008	4.020	82.729	107.012	123.005
2 - After	111.828	4.020	82.729	103.831	119.825

Note. Estimated means are estimated averaging across interacting variables

Trial

Trial	Mean	SE	df	95% Confidence Interval	
				Lower	Upper
Mean-1·SD	118.256	4.021	82.750	110.259	126.253
Mean	113.418	3.412	43.045	106.538	120.299
Mean+1·SD	108.580	4.021	82.751	100.583	116.578

Note. Estimated means are estimated averaging across interacting variables

FI

FI	Mean	SE	df	95% Confidence Interval	
				Lower	Upper
Mean-1·SD	111.619	4.825	43.057	101.888	121.349
Mean	113.418	3.412	43.045	106.538	120.299
Mean+1·SD	115.218	4.826	43.038	105.486	124.950

Note. Estimated means are estimated keeping constant other independent variable(s) in the model to the mean

Condition:Trial

Condition	Trial	Mean	SE	df	95% Confidence Interval	
					Lower	Upper
1 - Before	Mean-1·SD	118.756	5.022	196.479	108.852	128.659
2 - After	Mean-1·SD	117.757	5.021	196.381	107.855	127.658
1 - Before	Mean	115.008	4.020	82.729	107.012	123.005
2 - After	Mean	111.828	4.020	82.729	103.831	119.825
1 - Before	Mean+1·SD	111.261	5.017	195.799	101.367	121.155
2 - After	Mean+1·SD	105.900	5.026	197.064	95.989	115.810

Note. Estimated means are estimated keeping constant other independent variable(s) in the model to the mean

Fixed Effects Plots

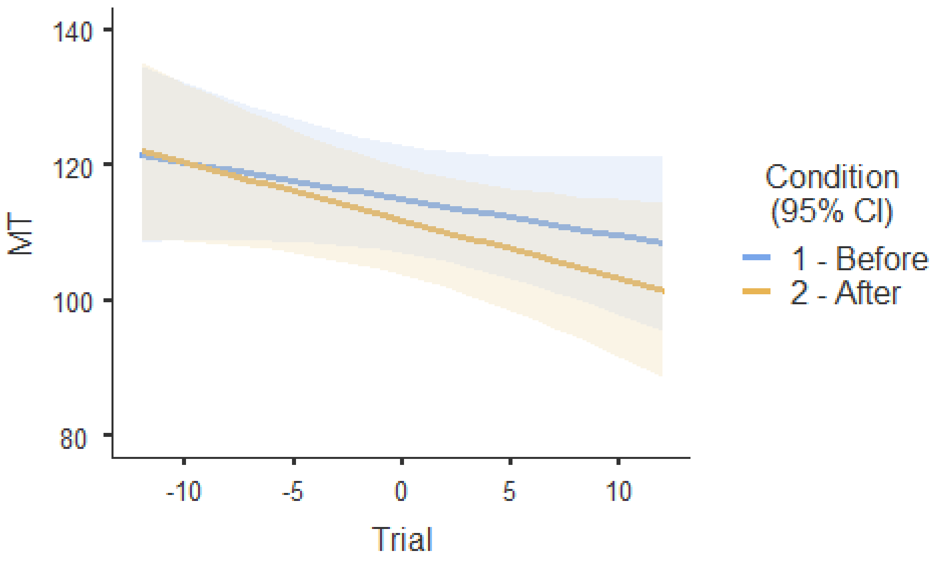

Mixed Model

Model Info

Info	
Estimate	Linear mixed model fit by REML
Call	Residuals RT ~ 1 + Condition + Trial + FI + Condition:Trial+(1|ID)
AIC	3000.430
R-squared Marginal	0.012
R-squared Conditional	0.141

Model Results

Fixed Effect Omnibus tests

	F	Num df	Den df	p
Condition	11.969	1	2101.870	< .001
Trial	0.724	1	2101.864	0.395
FI	1.969	1	42.805	0.168
Condition ✻ Trial	0.813	1	2101.867	0.367

Note. Satterthwaite method for degrees of freedom

Fixed Effects Parameter Estimates

Names	Effect	Estimate	SE	95% Confidence Interval Lower	Upper	df	t	p
(Intercept)	(Intercept)	0.477	0.031	0.416	0.537	53.896	15.465	< .001
Condition1	2 - After - 1 - Before	0.070	0.020	0.031	0.110	2101.870	3.460	< .001
Trial	Trial	-0.003	0.002	-0.007	0.001	2101.835	-1.240	0.215
FI	FI	-0.005	0.003	-0.011	0.002	42.805	-1.403	0.168
Condition1 ✻ Trial	2 - After - 1 - Before ✻ Trial	0.003	0.003	-0.003	0.008	2101.867	0.902	0.367

Random Components

Groups	Name	SD	Variance	ICC
ID	(Intercept)	0.183	0.033	0.131
Residual		0.472	0.222	

Note. Numer of Obs: 2150 , groups: ID , 45

Post Hoc Tests

Post Hoc Comparisons - Condition

Comparison Condition		Comparison Condition	Difference	SE	test	df	$p_{bonferroni}$
1 - Before	-	2 - After	-0.070	0.020	-3.460	2102.067	< .001

Estimated Marginal Means

Condition

Condition	Mean	SE	df	95% Confidence Interval Lower	Upper
1 - Before	0.477	0.031	53.896	0.415	0.538
2 - After	0.547	0.031	53.896	0.485	0.609

Note. Estimated means are estimated averaging across interacting variables

Trial

Trial	Mean	SE	df	95% Confidence Interval Lower	Upper
Mean-1·SD	0.520	0.031	53.901	0.459	0.582
Mean	0.512	0.029	42.804	0.453	0.570
Mean+1·SD	0.503	0.031	53.902	0.441	0.565

Note. Estimated means are estimated averaging across interacting variables

FI

FI	Mean	SE	df	95% Confidence Interval Lower	Upper
Mean-1·SD	0.553	0.041	42.808	0.470	0.635
Mean	0.512	0.029	42.804	0.453	0.570
Mean+1·SD	0.471	0.041	42.801	0.388	0.554

Note. Estimated means are estimated keeping constant other independent variable(s) in the model to the mean

Condition:Trial

Condition	Trial	Mean	SE	df	95% Confidence Interval Lower	Upper
1 - Before	Mean-1·SD	0.494	0.034	79.777	0.427	0.562
2 - After	Mean-1·SD	0.546	0.034	79.755	0.479	0.614
1 - Before	Mean	0.477	0.031	53.896	0.415	0.538
2 - After	Mean	0.547	0.031	53.896	0.485	0.609
1 - Before	Mean+1·SD	0.459	0.034	79.629	0.391	0.526
2 - After	Mean+1·SD	0.547	0.034	79.903	0.480	0.615

Note. Estimated means are estimated keeping constant other independent variable(s) in the model to the mean

Fixed Effects Plots

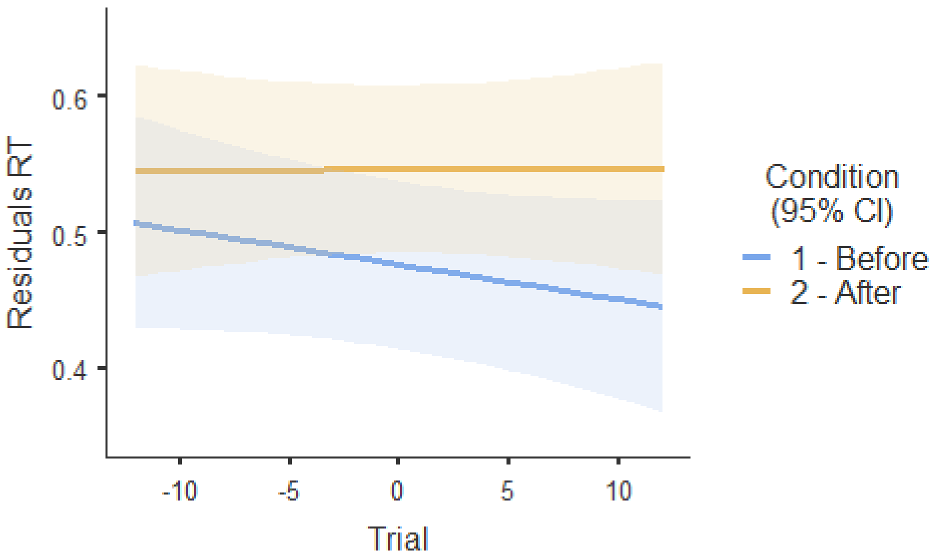

Mixed Model

Model Info

Info	
Estimate	Linear mixed model fit by REML
Call	Residuals MT ~ 1 + Condition + Trial + FI + Condition:Trial+(1|ID)
AIC	5643.318
R-squared Marginal	0.009
R-squared Conditional	0.050

Model Results

Fixed Effect Omnibus tests

	F	Num df	Den df	p
Condition	0.120	1	2102.028	0.729
Trial	5.011	1	2102.013	0.025
FI	4.858	1	42.852	0.033
Condition ✻ Trial	1.709	1	2102.019	0.191

Note. Satterthwaite method for degrees of freedom

Fixed Effects Parameter Estimates

Names	Effect	Estimate	SE	95% Confidence Interval Lower	Upper	df	t	p
(Intercept)	(Intercept)	0.380	0.038	0.306	0.455	75.817	9.983	< .001
Condition1	2 - After - 1 - Before	-0.013	0.038	-0.088	0.061	2102.028	-0.346	0.729
Trial	Trial	-0.003	0.004	-0.010	0.005	2101.933	-0.659	0.510
FI	FI	0.008	0.004	9.395e-4	0.016	42.852	2.204	0.033
Condition1 ✻ Trial	2 - After - 1 - Before ✻ Trial	-0.007	0.005	-0.018	0.004	2102.019	-1.307	0.191

Random Components

Groups	Name	SD	Variance	ICC
ID	(Intercept)	0.181	0.033	0.040
Residual		0.882	0.778	

Note. Numer of Obs: 2150 , groups: ID , 45

Post Hoc Tests

Post Hoc Comparisons - Condition

Comparison Condition		Condition	Difference	SE	test	df	$p_{bonferroni}$
1 - Before	-	2 - After	0.013	0.038	0.346	2102.180	0.729

Estimated Marginal Means

Condition

Condition	Mean	SE	df	95% Confidence Interval Lower	Upper
1 - Before	0.380	0.038	75.817	0.305	0.456
2 - After	0.367	0.038	75.816	0.291	0.443

Note. Estimated means are estimated averaging across interacting variables

Trial

Trial	Mean	SE	df	95% Confidence Interval	
				Lower	Upper
Mean-1·SD	0.416	0.038	75.834	0.341	0.492
Mean	0.374	0.033	42.847	0.307	0.441
Mean+1·SD	0.331	0.038	75.834	0.255	0.407

Note. Estimated means are estimated averaging across interacting variables

FI

FI	Mean	SE	df	95% Confidence Interval	
				Lower	Upper
Mean-1·SD	0.301	0.047	42.858	0.207	0.395
Mean	0.374	0.033	42.847	0.307	0.441
Mean+1·SD	0.447	0.047	42.841	0.352	0.541

Note. Estimated means are estimated keeping constant other independent variable(s) in the model to the mean

Condition:Trial

Condition	Trial	Mean	SE	df	95% Confidence Interval	
					Lower	Upper
1 - Before	Mean-1·SD	0.398	0.047	167.269	0.306	0.490
2 - After	Mean-1·SD	0.435	0.047	167.190	0.343	0.527
1 - Before	Mean	0.380	0.038	75.817	0.305	0.456
2 - After	Mean	0.367	0.038	75.816	0.291	0.443
1 - Before	Mean+1·SD	0.363	0.047	166.722	0.271	0.455
2 - After	Mean+1·SD	0.300	0.047	167.738	0.208	0.392

Note. Estimated means are estimated keeping constant other independent variable(s) in the model to the mean

Fixed Effects Plots

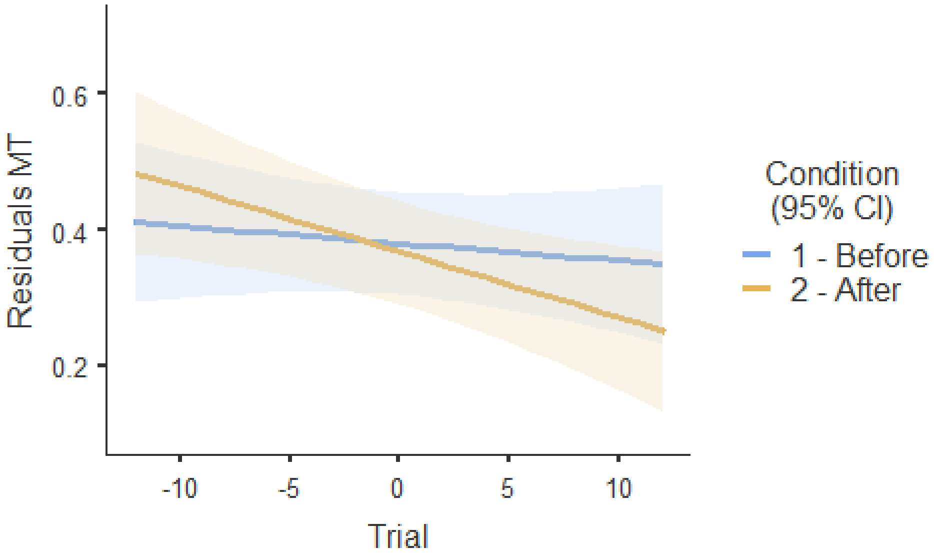

References

[1] The jamovi project (2019). *jamovi*. (Version 0.9) [Computer Software]. Retrieved from https://www.jamovi.org.

[2] R Core Team (2018). *R: A Language and envionment for statistical computing*. [Computer software]. Retrieved from https://cran.r-project.org/.
